# Database of open-framework aluminophosphate structures

**DOI:** 10.1038/s41597-020-0452-4

**Published:** 2020-03-31

**Authors:** Chuting Zheng, Yi Li, Jihong Yu

**Affiliations:** 1 State Key Laboratory of Inorganic Synthesis and Preparative Chemistry, College of Chemistry, Jilin University 2699 Qianjin Street, Changchun, 130012 China; 2 International Center of Future Science, Jilin University Changchun, 130012 China

**Keywords:** Cheminformatics, Solid-state chemistry

## Abstract

Open-framework aluminophosphates are an important class of inorganic crystalline compounds because of their rich structural chemistry and diverse properties. We have collected 312 open-framework aluminophosphate crystal structures from published literature and established a database for these structures. For each aluminophosphate structure, we have assigned a unique index code and extracted its key chemical and crystallographic information from the original literature and the associated CIF file, such as the name, chemical formula, extra-framework species, Al/P ratio, space group, and unit cell parameters of the compound. More importantly, we have calculated the topological features for each aluminophosphate framework, including local connectivity, framework dimension, coordination sequences, vertex symbols, topology density, and the largest ring. To help experimental chemists identify their products, we have also calculated theoretical XRD peaks for all aluminophosphate structures. This database will provide important insight into understanding the structural chemistry of open-framework aluminophosphate compounds.

## Abstract (summary)

**Table:** 

Measurement(s)	Chemical Structure • open-framework aluminophosphate
Technology Type(s)	digital curation • computational modeling technique

Machine-accessible metadata file describing the reported data: 10.6084/m9.figshare.11988558

## Background & Summary

Open-framework aluminophosphates^1^ are typically constructed from the alternate connection of Al-centred polyhedra (AlO_4_, AlO_5_ and AlO_6_) and PO_4_ tetrahedra by sharing bridging O atoms (μ-O)^2^. The frameworks of aluminophosphates can be one-, two-, or three-dimensionally extended, which normally crystallize together with extra-framework solvents, metal cations, or organic amine during their formation. Open-framework aluminophosphates have exhibited many attractive properties because of their featured framework structures and host-guest interactions^3^. In particular, AlPO zeolites, which are three-dimensional aluminophosphates constructed exclusively by AlO_4_ and PO_4_ tetrahedra, can be used for adsorption, ion-exchange, and catalysis after their extra-framework species are removed^4^. For instance, zeolite AlPO-LTA exhibits can be used in water-sorption-based energy storage and transformation because of its excellent hydrophilicity^5^; zeolite AlPO-18 can be fabricated as membranes, which can be used for CO_2_/CH_4_^6^ and Kr/Xe separation^7^. Meanwhile, some of the Al or P sites in AlPO zeolites can be occupied by heteroatoms, such as divalent metal or silicon atoms, providing aluminophosphate zeolites with tunable acidity and accompanying chemical properties. For instance, zeolite SAPO-34, in which parts of the P atoms in AlPO-34 are substituted by Si atoms, is an excellent industrial catalyst for methanol-to-olefin conversions^8,9^.

Understanding the rich structural chemistry of open-framework aluminophosphates is crucial to enhance their properties for practical applications. Because of their rich structure diversity, it is necessary to establish a database to collect all open-framework aluminophosphate structures and provide important structure information for users to investigate. To this end, we established a structure database for open-framework aluminophosphates in 2005, which collected about 200 aluminophosphate structures and 19 polyhedral connectivity types from published literature. This database has been widely used by many researchers in open-framework compounds^10–12^. Since then, the number of open-framework aluminophosphate structures has been growing rapidly. On the basis of our original database, we have now established a new database for open-framework aluminophosphate structures. In comparison with our original database, this new database contains 50% more structures and 100% more polyhedral connectivity types. More importantly, the new database contains more important structural features, which can be used not only to describe and to classify open-framework aluminophosphates but also to help people understand the structural chemistry of these compounds.

Currently, our new database contains 312 open-framework aluminophosphate structures, which have all been unambiguously determined by X-ray diffraction techniques. Besides charge-neutral AlPO zeolites composed of AlO_4_ and PO_4_, our database contains a large number of anionic frameworks with AlO_5_, AlO_6_, or interrupted structures. To describe these structures in details, we provide many types of structure attributes in this database, including chemical attributes, crystallographic attributes, and topological attributes. Chemical attributes include the index code we assign for each structure, the name and chemical formula of the compound, extra-framework species, and the Al/P ratio of the framework; crystallographic attributes include space group, unit cell parameters, atomic coordinates (the atom labelling is consistent with that reported in the original reference), the residual factor for XRD structure refinement (*R*_1_ for single-crystal data and *R*_wp_ for powder data)^13^ and the simulated XRD peak positions; topological attributes include the framework type code, polyhedral connectivity, framework dimension, coordination sequences, vertex symbols, topology density, and the largest ring.

To the best of our knowledge, this is the only structure database specialized for open-framework aluminophosphates, and most of the structure information in this database cannot be found in any other crystal structure database or even in their original literature. Using this database, theoretical chemists may easily find out the structure variation among different open-framework aluminophosphates and obtain insight in the structural chemistry of these compounds; experimental chemists may review all aluminophosphates that have been discovered and decide their synthetic targets for specific applications; crystallographers may search this database to identify their samples for structures with similar cell parameters. More importantly, this database provides various types of important structure data, which may serve as the input for future machine learning studies to reveal the complicated relationship among the structures, syntheses, and properties of open-framework aluminophosphates.

## Methods

### Data collection

All crystal structures were derived from the CIF files or the atomic coordinates reported in the original literature. Only structures that were unambiguously determined using X-ray diffraction techniques were collected in our database. Before adding a new structure into our database, we checked the difference between the new structure and those already included in our database. Only structures significantly different from existing ones were added. In the end, we obtained a total of 312 open-framework aluminophosphate structures. For each structure, we assigned a unique code in the format of “*d.a.p.spg.sn*”, where *d* is the dimension of the framework, *a* and *p* represent the Al/P ratio in coprime integers, *spg* is the sequence number of its space group defined in the Internal Tables For Crystallography^14^, and *sn* is a serial number that discriminates different structures in our database.

### Structure attribute calculation

Most of the chemical and crystallographic attributes were extracted from the source literature and the CIF files associated. XRD simulations were performed using Materials Studio^15^. Topological attributes, such as coordination sequences, vertex symbols, the largest ring and TD10 calculations, were calculated using ToposPro^16^.

Simulated XRD peaks. To help phase identification, the powder XRD peaks were simulated for each structure according to its crystal structure. XRD peaks were listed in the descending order of d-spacing. In addition, the peak positions of the strongest three reflections were listed explicitly in 2*θ* degrees assuming Cu *K*α1 radiation.

Coordination sequences consist of sequences of integers, reflecting the numbers of neighbouring atoms in different coordination shells of specific central atoms. Coordination sequences can be used to distinguish different framework topologies.

Vertex symbols indicate the sizes of the smallest rings associated with the coordination angles of the central atom. For a central atom connected with *n* neighbours, the vertex symbols consist of *n* × (*n* − 1)/2 integers. If no ring was found for an angle, the vertex symbol would be indicated by an asterisk.

Largest ring. We calculated the number of polyhedral central atoms involved the largest ring.

TD10 is defined as the average number of neighbouring atoms in the first ten coordination shells of specific central atoms in a framework structure, which reflects the topological density of the framework.

## Data Records

**Fig. 1:**
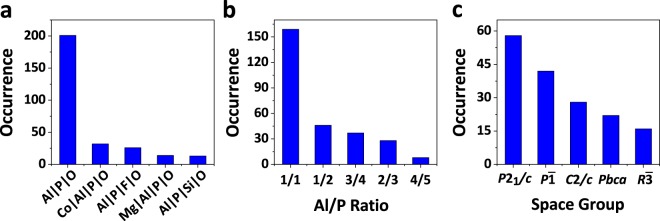
Occurrence of top-five (**a**) framework compositions, (**b**) Al/P ratios, and (**c**) space groups in our open-framework aluminophosphate database.

Currently, our database consists of 312 aluminophosphate structures, which can be accessed at figshare^17^. Among these structures, 201 are pure aluminophosphates, the frameworks of which are constructed exclusively by Al, P, and O; 72 structures contain transition metals in their frameworks, 27 structures contain fluorine, and 15 structures contain silicon. Figure 1a shows the occurrence of the top-five framework compositions in our database.

The Al/P ratio is an important structural feature for open-framework aluminophosphates, which determines the electronegativity of the framework. In our database, there are 159 structures with Al/P ratios of 1.0, most of which correspond to neutral frameworks. 146 structures exhibit Al/P ratios less than 1.0, corresponding to anionic frameworks. Among these anionic structures, Al/P ratios of 1/2, 2/3, 3/4 and 4/5 are the most frequently observed (Fig. 1b). Moreover, there are 7 structures exhibiting Al/P ratios more than 1.0, containing rarely observed cationic frameworks.

From the symmetry’s point of view, the aluminophosphate structures in our database belong to 70 space groups, covering more than 1/3 of all possible space groups. Low-symmetry space groups are much more common than high-symmetry ones for open-framework aluminophosphates. In particular, structures in space groups *P*2_1_/*c*, *P*$\bar{1}$, *C*2*/c*, *Pbca*, and *R*$\bar{3}$ amount to over half of our database. These space groups are also among the most frequently observed ones in other crystal structure database^18^ (Fig. 1c).

203 structures in our database exhibit three-dimensional frameworks, constituting nearly 2/3 of our database. Besides, 85 structures exhibit two-dimensional layered frameworks, and 23 exhibit one-dimensional chained frameworks. In particular, we include a zero-dimensional structure, (N_2_C_2_H_10_)_4_(NH_4_)AlP_4_O_16_ (Index code: 0.1.4.82.001), which consists of a pentameric cluster of one AlO_4_ and four PO_4_ tetrahedra^19^.

**Fig. 2:**
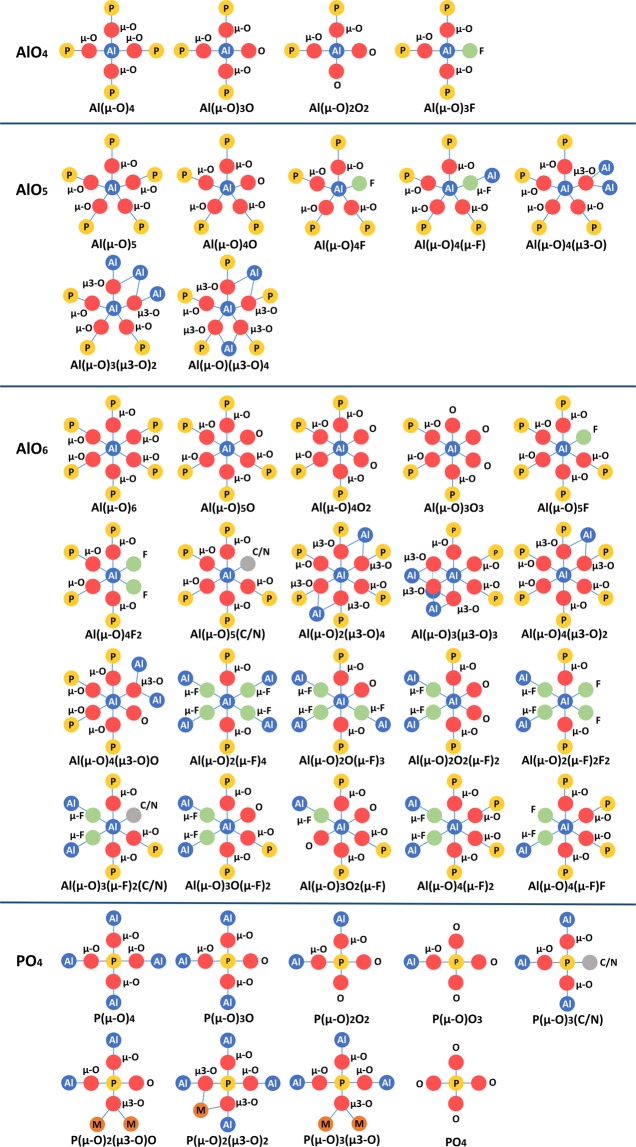
All possible polyhedral connectivity observed in open-framework aluminophosphate database. Blue, yellow, orange, red, green and grey circles represent Al, P, transition metals, O, F, and templating agents, respectively.

Although all the P atoms in open-framework aluminophosphates are 4-coordinated, the Al atoms can be 4, 5, or 6-coordinated. More importantly, the connectivity of Al and P is even more diverse than their coordination states, which leads to the rich structure diversity of open-framework aluminophosphates (Fig. 2). For instance, 4-coordinated P atoms can be 1-, 2-, 3-, and 4-connected to neighbouring Al atoms via bridging O atoms; 4-coordinated Al atoms can be 2-, 3- and 4-connected to neighbouring P atoms via bridging O atoms; 5-coordinated Al atoms can be 4- and 5-connected to neighbouring P or Al atoms via O or F bridges; 6-coordinated Al atoms can be 3-, 4-, 5-, and 6-connected to neighbouring P or Al atoms via O or F bridges. On the other hand, an O or F atom may be the bridge connecting one Al and one P atoms (μ-O/F), connecting two Al and one P atoms (μ3-O), or connecting three Al atoms (μ3-O); it may also connect to only one Al or P atom (O/F). Considering the difference between O and F bridges and the existence of heteroatoms other than Al and P, the number of connectivity types for polyhedra in open-framework aluminophosphates has reached 40. Figure 2 shows all of the 40 types of polyhedral connectivity. Currently, UiO-26-as (Index code: 3.1.1.14.008) exhibits the most diverse polyhedral connectivity in our database, which contains 4-, 5-, 6-coordinated Al and five types of connectivity in its three-dimensional framework, including Al(μ-O)_4_, Al(μ-O)_3_(μ3-O)_2_, Al(μ-O)_4_(μ3-O), Al(μ-O)_5_O and P(μ-O)_4_^20^. In comparison, all zeolitic aluminophosphates contain Al(μ-O)_4_ and P(μ-O)_4_ exclusively, representing the simplest polyhedral connectivity in our database.

**Fig. 3:**
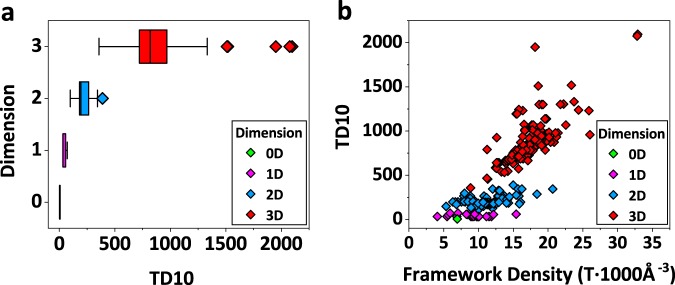
(**a**) TD10 for structures of different framework dimensions, and (**b**) correlation between TD10 and framework density.

Coordination sequences and TD10 are both the numbers of neighbouring atoms in the first few coordination shells from specific central atoms, reflecting the topological density of a framework structure. Coordination sequences are calculated for each crystallographically distinct atoms, whereas TD10 is the sum of all shells averaged among all distinct atoms. So TD10 is more “isotropic” than coordination sequences. Besides being used to distinguish different framework topologies, we can deduce important structure information from these topological attributes. For instance, we can deduce the dimension of an aluminophosphate framework according to its coordination sequences and TD10. Low-dimensional frameworks contain much more interrupted P or Al sites than high-dimensional frameworks, which will stop the rapid growth of the number of neighbouring atoms and break the connectivity of the framework structure. Figure 3a shows the distribution of TD10 in structures of different framework dimensions. For three-dimensional aluminophosphate frameworks, TD10 ranges from 357 to 2096, and the median TD10 is 818 in our database. For two-dimensional frameworks, TD10 varies in a much narrower range between 99 to 388, and the median TD10 for two-dimensional frameworks is 194, much lower than that of three-dimensional frameworks. TD10 for one-dimensional frameworks varies in an even narrower and lower range than that of two-dimensional frameworks (Fig. 3a). The ranges of TD10 among different framework dimensions almost do not overlap, so we can use TD10 to estimate the framework dimension. Figure 3b shows the plot of TD10 versus the framework density for all structures in our database. The correlation coefficient R^2^ is 0.72393, indicating a significant correlation between TD10 and framework density. Therefore, TD10 can not only reflect the topological density, but also the general trend of framework density.

**Fig. 4:**
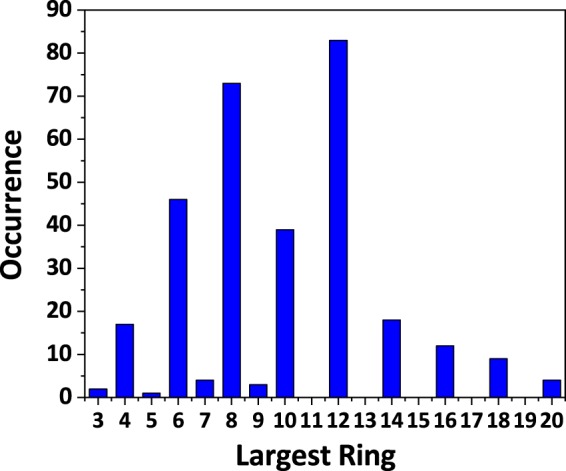
Distribution of the largest ring in open-framework aluminophosphate database.

The porosity of aluminophosphate structures can be described by the rings in aluminophosphate frameworks. In this database, the rings are described using vertex symbols, which reflect the sizes of the rings passing the same central atoms. In addition, we introduce the largest ring to describe the largest pore opening in each aluminophosphate framework. Figure 4 shows the distribution of the largest ring in our database. 12-, 8-, 6-, and 10-rings are the most frequently observed largest rings among all open-framework aluminophosphates. Odd-numbered rings are much fewer than even-numbered rings in open-framework aluminophosphate structures, which may result from the fact that alternate linkage of AlO_4_ and PO_4_ tetrahedra is highly favoured over direct linkage of two AlO_4_ tetrahedra according to the Löwenstein’s Rule^21^. So far, only 10 open-framework aluminophosphate structures contain odd-numbered rings as the largest pore openings in their frameworks. For instance, three-dimensional AlPO-CJ3 (Index code: 3.1.1.61.008) contains nine-membered rings formed by three AlO_6_ octahedra, two AlO_4_ and four PO_4_ tetrahedra^22^, and one-dimensional chained structure AlPO-CJ10 (Index code: 1.1.2.62.001) contains three-membered rings formed by two AlO_6_ octahedra and one PO_4_ tetrahedron^23^.

**Fig. 5:**
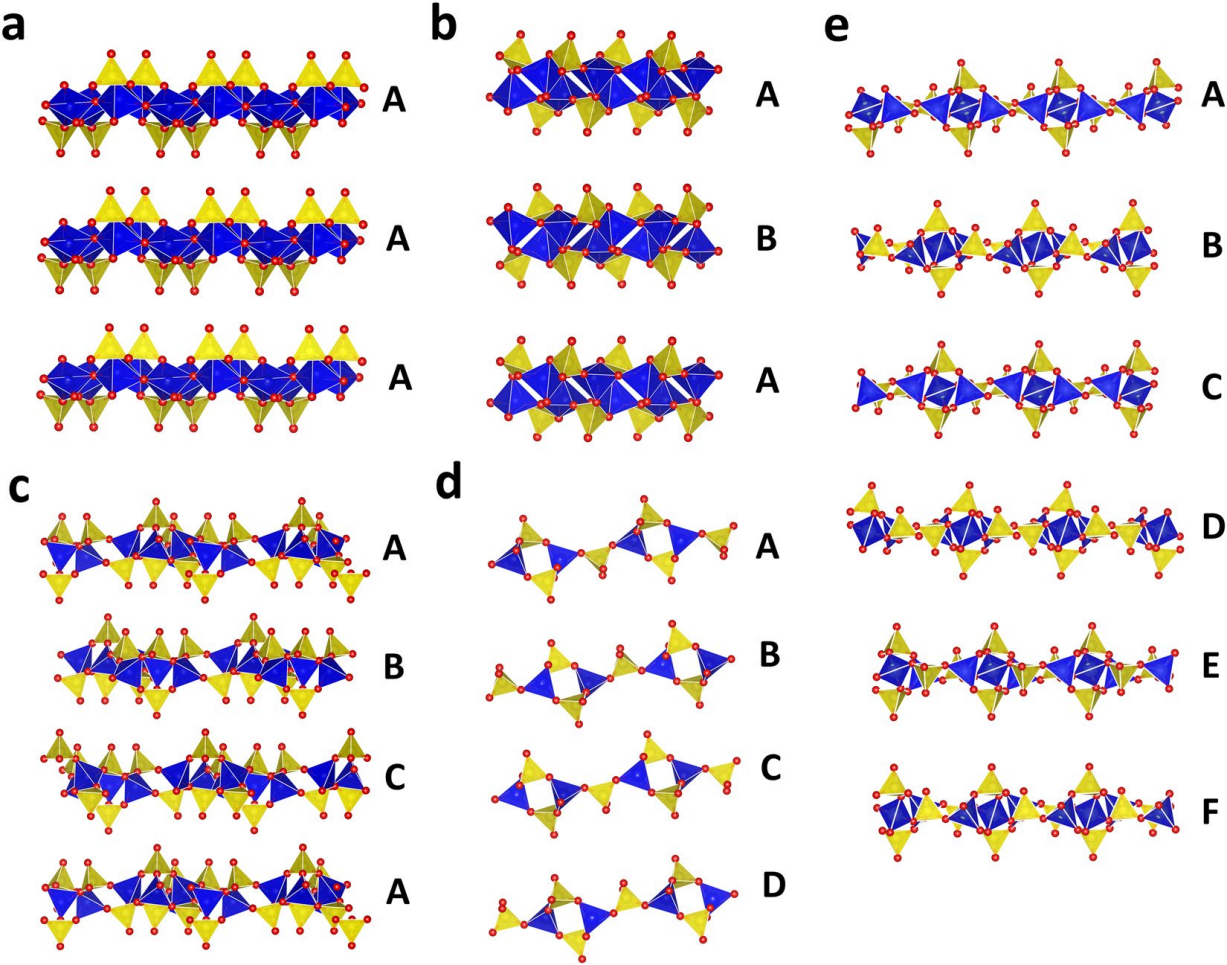
Layer stacking in (**a**) UiO-15-125^24^, (**b**) UiO-18-100^25^, (**c**) [NH_3_CH_2_CH(OH)CH_3_]_3_[Al_3_P_4_O_16_]^26^, (**d**) (C_2_H_8_N)_2_[Al_2_(HPO_4_)(PO_4_)_2_]^27^, and (**e**) [Co(C_4_N_3_H_13_)_2_][Al_3_P_4_O_16_][3H_2_O]^28^. Blue and yellow polyhedra represent Al- and P-centred polyhedra; red spheres represent O atoms. Extra-framework species are omitted for clarity.

For two-dimensional layered aluminophosphates, the stacking sequences of the layers are important for the construction of the complete crystal structures. In our database, there are five types of stacking sequences for aluminophosphate layers, namely AA, AB, ABC, ABCD, and ABCDEF (Fig. 5). In structures with AA stacking, such as UiO-15-125^24^ (Index code: 2.1.1.14.001), each layer can overlap with adjacent layers by simple translation (Fig. 5a). For other stacking manners, layers cannot overlap with each other by simple translation. For instance, in UiO-18-100^25^ (Index code: 2.1.1.14.003), every pair of adjacent layers overlap with each other by rotating for 180° along the 2_1_ axis parallel to the layers, so the stacking sequence is AB (Fig. 5b); in [NH_3_CH_2_CH(OH)CH_3_]_3_[Al_3_P_4_O_16_]^26^ (Index code: 2.3.4.148.001), adjacent layers overlap with each other by rotating for 120° along the 3_1_ axis perpendicular to the layers, so the stacking sequence is ABC (Fig. 5c); in (C_2_H_8_N)_2_[Al_2_(HPO_4_)(PO_4_)_2_]^27^ (Index code: 2.2.3.15.001), every layer overlaps with adjacent layers either by rotating for 180° along the 2_1_ axis parallel to the layers or by inverse toward the inversion centre between adjacent layers, so the stacking sequence is ABCD (Fig. 5d); in [Co(C_4_N_3_H_13_)_2_][Al_3_P_4_O_16_][3H_2_O]^28^ (Index code: 2.3.4.179.001), adjacent layers overlap with each other by rotating for 60° along the 6_1_ axis perpendicular to the layers, so the stacking sequence is ABCDEF (Fig. 5e). In our database, AA- and AB-stacking structures are the most frequently observed, amounting to 90% of all layered structures in our database.

## Technical Validation

Most structure attributes in our database were calculated by Materials Studio and ToposPro, which are computer programs widely used by researchers from all over the world. We have also compared our data with those obtained from other sources, such as the online structure database of the International Zeolite Association^4^, to verify the accuracy of our data. Selected comparison results are listed in Tables 1 and 2.

**Table 1:** Comparison of the coordination sequences of selected aluminophosphate structures between our database and the online database of the International Zeolite Association (IZA).

Index code	IZA code	Coordination sequences (IZA)	Coordination sequences (open-framework aluminophosphate database)
3.1.1.15.002	AEI	T1: 4 9 17 29 45 64 85 111 143 177 T2: 4 9 17 29 45 65 88 113 143 178 T3: 4 9 17 29 45 65 87 113 143 176	Al1(P1): 4 9 17 29 45 64 85 111 143 177 Al2(P2): 4 9 17 29 45 65 88 113 143 178 Al3(P3): 4 9 17 29 45 65 87 113 143 176
3.1.1.46.001	AEL	T1: 4 11 21 37 59 85 114 150 189 232 T2: 4 11 22 38 58 85 115 148 188 234 T3: 4 12 24 40 59 84 115 150 186 230	Al1(P1): 4 11 21 37 59 85 114 150 189 232 Al2(P2): 4 11 22 38 58 85 115 148 188 234 Al3(P3): 4 12 24 40 59 84 115 150 186 230
3.1.1.36.001	AET	T1: 4 12 23 36 52 75 103 135 172 215 T2: 4 11 22 38 55 74 98 132 173 216 T3: 4 11 21 35 53 78 108 140 172 208 T4: 4 11 21 35 52 74 102 136 172 212 T5: 4 10 18 32 52 76 105 140 171 202	Al1(P1): 4 12 23 36 52 75 103 135 172 215 Al2(P2): 4 11 22 38 55 74 98 132 173 216 Al3(P3): 4 11 21 35 53 78 108 140 172 208 Al4(P4): 4 11 21 35 52 74 102 136 172 212 Al5(P5): 4 10 18 32 52 76 105 140 171 202
3.1.1.148.003	CHA	T1: 4 9 17 29 45 64 85 110 140 173	Al1(P1): 4 9 17 29 45 64 85 110 140 173
3.1.1.184.003	AFI	T1: 4 11 21 35 53 77 105 137 172 212	Al1(P1): 4 11 21 35 53 77 105 137 172 212
3.1.1.148.002	SAT	T1: 4 10 20 33 50 71 95 124 158 197 T2: 4 9 17 30 50 75 100 126 157 194	Al1(P1): 4 10 20 33 50 71 95 124 158 197 Al2(P2): 4 9 17 30 50 75 100 126 157 194

**Table 2:** Comparison of the vertex symbols of selected aluminophosphate structures between our database and the online database of the International Zeolite Association (IZA).

Index code	IZA code	Vertex symbols (IZA)	Vertex symbols (open-framework aluminophosphate database)
3.1.1.15.002	AEI	T1: 4.4.4.8.6.8 T2: 4.4.4.8.6.8 T3: 4.4.4.8.6.8	Al1(P1): 4.4.4.8.6.8 Al2(P2): 4.4.4.8.6.8 Al3(P3): 4.4.4.8.6.8
3.1.1.46.001	AEL	T1: 4.6(2).6.6(3).6(2).6(3) T2: 4.6(2).6.6(3).6(2).6(3) T3: 6.6(2).6(2).6(2).6(2).6(2)	Al1(P1): 4.6(2).6.6(3).6(2).6(3) Al2(P2): 4.6(2).6.6(3).6(2).6(3) Al3(P3): 6.6(2).6(2).6(2).6(2).6(2)
3.1.1.36.001	AET	T1: 6.6(2).6(2).6(2).6(2).6(2) T2: 4.6(2).6.6(3).6(2).6(3) T3: 4.6(2).6.6(3).6(2).6(3) T4: 4.6(2).6(2).6(3).6(2).6(3) T5: 4.6(3).4.6(3).6.6(4)	Al1(P1): 6.6(2).6(2).6(2).6(2).6(2) Al2(P2): 4.6(2).6.6(3).6(2).6(3) Al3(P3): 4.6(2).6.6(3).6(2).6(3) Al4(P4): 4.6(2).6(2).6(3).6(2).6(3) Al5(P5): 4.6(3).4.6(3).6.6(4)
3.1.1.148.003	CHA	T1: 4.4.4.8.6.8	Al1(P1): 4.4.4.8.6.8
3.1.1.184.003	AFI	T1: 4.6(2).6.6(3).6(2).6(3)	Al1(P1): 4.6(2).6.6(3).6(2).6(3)
3.1.1.148.002	SAT	T1: 4.6.4.8.6.6 T2: 4.4.4.6.6.8	Al1(P1): 4.6.4.8.6.6 Al2(P2): 4.4.4.6.6.8

## Data Availability

ToposPro (version 5.0) is available at http://topospro.com; Materials Studio 2018 (version 18.1.0.2017) is a commercial program package, which is available at BIOVIA.
